# Expression of Concern for The Effect of Dexmedetomidine and Esmolol on Early Postoperative Cognitive Dysfunction After Middle Ear Surgery Under Hypotensive Technique: A Comparative, Randomized, Double-blind Study [Anesth Pain Med. 2021;11(1): e107659]

**DOI:** 10.5812/aapm.148635

**Published:** 2024-05-07

**Authors:** Mahmood-Reza Alebouyeh

**Affiliations:** 1 Iran University of Medical Sciences, Tehran, Iran

Dear Readers,

We, the editorial team of AAPM, are committed to upholding the highest standards of academic integrity and ethical publishing practices. It is with this commitment in mind that we address the current situation regarding the mentioned article (1) recently published as inpress in our journal.

Recently, we received a concerning claim (#524055) from a whistleblower regarding potential misconduct related to the data and content of the article. As custodians of scholarly integrity, we take such claims seriously and have initiated a thorough investigation into the matter. 

In light of these allegations and respecting our policy about allegations of misconduct (link), we are conducting a comprehensive review to ascertain the validity of the claims and ensure that the integrity of the published research is maintained.

As we navigate through this process, we remain committed to transparency and will provide updates on the investigation's progress and outcomes. We understand the importance of promptly addressing concerns related to research integrity and appreciate your patience and understanding as we work to uphold the principles of ethical publishing.

Should you have any questions or concerns regarding this matter, please do not hesitate to reach out to us. Your trust and confidence in the integrity of our journal are of utmost importance to us, and we are dedicated to maintaining the highest standards of scholarly publishing ethics.

Sincerely,

Head of Ethics Committee, Brieflands

On Behalf of Editorial Team of AAPM

References
